# Spray Method—What It Can Do for Catarrh and Its Effects

**Published:** 1886-02-20

**Authors:** Hiram Christopher

**Affiliations:** Prof. of Chemistry in the St. Joseph Medical College, St. Joseph, Mo.


### THE SPRAY METHOD. WHAT IT CAN DO FOR CATARRH AND ITS EFFECTS.

BY HIRAM CHRISTOPHER, A. M., M. D.,
**Prof. of Chemistry in the St. Joseph Medical College, St. Joseph, Mo.**

In almost every newspaper there are to be seen advertisements of catarrh cures, all of which, though differing as widely as the poles of the earth are distant from each, are nevertheless infallible cures; and even people, intelligent in other matters, are found putting such faith in them as to give them a trial. The trial ends in failure and oftentimes in injury; but these are never advertised as a warning or a caution to other sufferers.

Then again, catarrhal patients apply to their doctors—general practitioners—who suggest some simple remedy in order to be clear of the patient; who, really knowing nothing of the disease nor of the proper method and means of treatment, encourage by their failures trials of advertised remedies; so that between the two, no one is cured, or even much relieved of the troublesome disease; and the report is spread far and wide that no one can cure catarrh. When, therefore, one who knows says that it can be and has been cured, his word is discredited, or persons imagine that their money will go the way of its predecessor spent on advertisements, if they give the specialist a trial. And so they end in doing nothing. It is not unfrequently asked, have you ever cured anyone? Can catarrh be cured? Can I be made to hear as well as ever?

Following an illustrious examplar, one who replied to a leading question "Go, and tell the questioner what you see and hear," I submit a few cases, typical of many, to show what has been, and can be, done.

CASE I.

A little girl eight years old, of light hair, blue eyes, not robust, of mixed temperament, of a somewhat strumous diathesis, was brought in July, 1883. She had been suffering from nasal catarrh for more than a year, and had been under constant treatment the whole of the time, without any benefit. At the time of her coming, both nasal passages were plugged to the external orifice by hardened secretions. The patient breathed entirely by the mouth and had been doing so for several months. The throat was but little affected. There was a slight enlargement of the tonsils.

The treatment was begun on the 10th of July and continued to the 17th, when the patient went on a visit to Iowa with her mother. At this time she was sleeping with her mouth open. Treatment was again commenced on the 1st of August and continued for four days, renewed again on the 23rd, and thence continued through September daily, and the fall at intervals. She was well in October, but it was deemed prudent to keep up the treatment at intervals. There was a steadv improvement from the beginning. No return at this date, August, 1885.

CASE II.

Another little girl, age 6, about the same time, who presented an opposite case, Here there was profuse discharge from the nose of muco-purulent matter, with some impairment of hearing in the left ear. The patient was slight and delicate in form, brown eyes and brown hair. The throat but slightly involved: slight enlargement of the tonsils. The treatment was continued, daily at first and then at intervals, for three weeks, when she was dismissed well. There was no return until this spring, when a few treatments relieved her fully.

CASE III.

A young man, a farmer, about 30. Had been suffering from nasal catarrh for over a year. The discharge was somewhat copious and offensive. Throat considerably inflamed, but this soon disappeared under general local treatment. In this case, vaseline and resorcin were used, 6 grains of the latter to one ounce in volume of the former. The treatment was begun on the 20th of Sept. and contined daily through the month, and at intervals during the fall, and terminated on the first of Jan. following. In this case the resorcin removed all odor in a few treatments, and has been used in like cases since with like excellent results. This party continued sound until within the last month—July, 1885—when, after a few treatments, he was again relieved, expressing himself as perfectly satisfied that the spray method does cure.

### CASE IV.

A case of pharyngo-nasal catarrh attended by deafness in the right ear. A young lady, about 26, stoutly built, blue eyes and brown hair, and of fair complexion, when in health. Had scarlet fever in childhood, which was followed by slight deafness and frequent ear-aches in the left ear. Heard well in the right, but became suddenly deaf in this ear some four years ago. Found herself deaf on rising in the morning. Thinks she had had cattarrh from childhood, the discharge being generally copious and free. Had been treated for the deafness in Montana by Politzer's method only, without benefit. Had at the time of applying a slight discharge from the right meatus. She could not hear ordinary conversation, nor the organ in her church. Watch heard only on contact. Her general condition was bilious, but otherwise fair health. This condition was soon relieved by daily doses of the mercu. chlo. 1 gr. pill doses. The pharyngo-nasal inflammation was treated by the usual spray-method, and the eustachian tubes opened by inflammation by Politzer's method. The treatment was begun May 2nd, 1884, and continued daily and at intervals until September, when she could hear ordinary conversation while walking with friends on the street, and at home. She was treated once this month—August, 1885—because of fresh cold. She continues to hear well.

### CASE V.

A young lady, aged 19. Deaf in both ears, watch only heard on contact. General health fair. Has been deaf for some six years. Has pharyngo-nasal catarrh, not profuse nor obstructive to respiration through the nasal passages. Treated daily for three weeks and then on alternate days for a week. Lives in an adjoining county, and left for home at the end of a month, hearing distinctly the bubbling in the spray-bulb when cooling the vaseline two feet distant and ordinary conversation in the sitting room. This case will require treatment in the fall, because of its long duration.

These cases, a few out of many, are sufficient to show what hope may be entertained by those afflicted in this way. The good heart rejoices that such sufferings can be relieved.—*St. Louis Medical Journal.*

